# The effects of normobaric and hypobaric hypoxia on cognitive performance and physiological responses: A crossover study

**DOI:** 10.1371/journal.pone.0277364

**Published:** 2022-11-10

**Authors:** Erich Hohenauer, Livia Freitag, Joseph T. Costello, Thomas B. Williams, Thomas Küng, Wolfgang Taube, Miriam Herten, Ron Clijsen

**Affiliations:** 1 Rehabilitation and Exercise Science Laboratory (RES lab), Department of Business Economics, Health and Social Care, University of Applied Sciences and Arts of Southern Switzerland, Landquart, Switzerland; 2 International University of Applied Sciences THIM, Landquart, Switzerland; 3 Department of Neurosciences and Movement Science, University of Fribourg, Fribourg, Switzerland; 4 Department of Movement and Sport Sciences, Vrije Universiteit Brussel, Brussels, Belgium; 5 Extreme Environments Laboratory, School of Sport, Health and Exercise Science, University of Portsmouth, Portsmouth, United Kingdom; 6 Department of Health, Bern University of Applied Sciences, Berne, Switzerland; Tokyo Joshi Ika Daigaku Toyo Igaku Kenkyujo Clinic, JAPAN

## Abstract

This partially randomised controlled, crossover study sought to investigate the effects of normobaric hypoxia (NH) and hypobaric hypoxia (HH) on cognitive performance, the physiological response at rest and after a 3-min step-test. Twenty healthy participants (10 females and 10 males, 27.6±6.2yrs, 73.6±13.7kg, 175.3±8.9cm) completed a cognitive performance test, followed by the modified Harvard-step protocol, in four environments: normobaric normoxia (NN; P_i_O_2_: 146.0±1.5mmHg), NH (P_i_O_2_: 100.9±1.3mmHg), HH at the first day of ascent (HH1: P_i_O_2_ = 105.6±0.4mmHg) and HH after an overnight stay (HH2: P_i_O_2_ = 106.0±0.5mmHg). At rest and/or exercise, SpO_2_, NIRS, and cardiovascular and perceptual data were collected. The cerebral tissue oxygenation index and the cognitive performance (throughput, accuracy, and reaction time) were not different between the hypoxic conditions (all p>0.05). In NH, SpO_2_ was higher compared to HH1 (ΔSpO_2_ NH vs HH1: 1.7±0.5%, p = 0.003) whilst heart rate (ΔHR NH vs HH2: 5.8±2.6 bpm, p = 0.03) and sympathetic activation (ΔSNSi NH vs HH2: 0.8±0.4, p = 0.03) were lower in NH compared to HH2. Heart rate (ΔHR HH1 vs HH2: 6.9±2.6 bpm, p = 0.01) and sympathetic action (ΔSNSi HH1 vs HH2: 0.9±0.4, p = 0.02) were both lower in HH1 compared to HH2. In conclusion, cognitive performance and cerebral oxygenation didn’t differ between the hypoxic conditions. SpO_2_ was only higher in NH compared to HH1. In HH2, heart rate and sympathetic activation were higher compared to both NH and HH1. These conclusions account for a P_i_O_2_ between 100–106 mmHg.

## Introduction

Normoxia is known to be the state in which there is an adequate supply of oxygen in the human tissues. In case of reduced oxygen supply to the tissue, the environment is said to be hypoxic [1]. Unique physiological findings under severe hypoxia resulted from the Silver hut expedition in the 1960s and were carried out under terrestrial altitude [2]. It is well known that sudden exposure to hypoxia affects several fundamental physiological systems, e.g., the cardiovascular and central nervous system [3, 4].

Hypoxic environmental conditions are typically found in the mountains, where the atmospheric partial pressure of oxygen decreases, proportionally with the reduction of the barometric pressure (P_B_) [5]. Technological progress nowadays allows research, in the field of hypoxia, to be conducted under normobaric conditions in the laboratory. The equivalent air altitude model stipulates that different combinations of P_B_ and O_2_ fraction, producing the same partial pressure of O_2_ (P_i_O_2_), result in similar physiological responses [6]. However, the physiological differences after exposure to normobaric hypoxia (NH) and hypobaric hypoxia (HH) are currently a topic of much debate [7, 8]. Millet and colleagues (2020) conclude from the available literature that P_B_ may independently exert an important influence on many physiological responses between normobaric and hypobaric hypoxia [7]. Conversely, Richalet and colleagues (2020) debated that a specific effect of P_B_ within the body is limited as there is no barometric sensor [8]. Additionally, Richalet and colleagues (2020) suggested that HH studies give the exact P_B_ and that studies in NH would provide the F_i_O_2_ and the exact P_B_ [8].

A meta-analysis of 13 studies comparing NH and HH reported several variables (e.g. minute ventilation and nitric oxide levels) were different, lending support to the notion that true physiological differences are indeed present [9]. However, there is some evidence to suggest that this is not the case for all physiological variables. For example, cerebral oxygenation [10] and heart rate variability [11] are similar following exposure to both NH and HH. Unfortunately, as highlighted by Coppell and colleagues (2015) the current evidence base limits the conclusions which can be drawn due to various confounding factors such as the duration of exposure, temperature, humidity, and small sample sizes [9].

In addition to the debated physiological differences following NH and HH, the impact of both environments on cognitive performance is also ambiguous. The human brain requires a continuous supply of oxygen to function effectively. Consequently, cognitive performance can be impaired due to acute hypoxic exposures, leading to possible issues in decision-making with potentially fatal consequences [12]. This is of interest to mountaineers, pilots and other individuals who are often exposed to hypoxic environments. Tasks, that include memory, attention and executive functions, often categorized as “simple” or “complex”, are used to measure cognitive performance [13, 14]. Few researchers have considered how alterations in physiological homeostasis may translate to cognitive performance [15]. It is well established that moderate to severe hypoxia can impair cognitive capabilities [16, 17]. What is comparatively less clear is whether or not P_B_ also plays a role. Measuring cerebral oxygenation, using near-infrared spectroscopy, is a non-invasive way to objectify local indices of oxygen deficits in cerebral regions [12, 18]. Therefore, it is important to consider cerebral oxygenation measurements to elucidate the mechanisms responsible for cognitive function under NH and hypobaric hypoxia HH. Previous meta-regression findings suggested that PaO_2_, and not whether the exposure was normobaric or hypobaric *per-se*, appears to be the key predictor of any decline in cognitive performance [17]. These findings are in line with results presented from another research group, demonstrating that the hypobaric effect is negligible on cognitive performance in hypoxia [4]. However, these findings were also limited due to the methodological quality, heterogeneous nature of the outcome measures reported and the number of participants included in the analysis (n = 437) [17]. Williams and colleagues (2019) have recently established that cerebral oxygenation, and peripheral oxygen saturation are correlated with cognitive performance during NH [19]. It is therefore plausible that the proposed similarities observed in cerebral oxygenation after both NH and HH may translate to similar findings in cognitive performance. However, this theory warrants further exploration.

Another important approach of investigation that needs further evaluation is the physiological response to exercise under NH and HH. Exercise under hypoxia has been demonstrated to substantially decrease arterial oxygen saturation compared to normoxic conditions [20, 21]. It has been reported that arterial oxygen saturation (SpO_2_) is lower during acute HH exposures [22, 23], which occurs probably due to increased dead-space ventilation in this condition [24]. However, these differences were not present during longer exposures to hypoxia, which can be attributed probably to acclimatisation processes [24, 25]. Interesting results were observed from a research group that investigated muscle oxygenation during an incremental cycling test. These authors observed that muscle oxygenation was higher in HH compared to NH and attributed these results to the higher cardiac output and to the higher hypoxic dose during HH [26]. To evaluate the impact of the reduced partial pressure of oxygen on the autonomic nervous system, heart rate variability (HRV) has been measured in previously published studies, reflecting the level of activity of the sympathetic and vagal components [27, 28]. Hypoxia has also been shown to decrease parasympathetic activity (HRV, high-frequency bands) in resting conditions [29]. It has been postulated, that HH represents a more severe environment leading to different physiological responses compared to NH [30, 31]. Published results have demonstrated, that HH led to greater reductions in maximal heart rate and consequently endurance performance, compared to NH [32, 33]. Conversely, no difference between NH and HH were observed regarding lactate concentrations after a cycle time-trial performance [33]. Data has also shown no difference between NH and HH for cortisol levels under resting conditions [34, 35] whilst others did find a difference [36]. In addition, when compared to NH, HH has also been demonstrated to lead to more pronounced sleep disturbances [37]. To our knowledge, there is only a limited number of studies, that have investigated the difference between NH and HH (HH1), and further compared them to an overnight stay in HH (HH2).

Accordingly, using a partially randomized controlled, crossover design, this study sought to investigate the effects of both NH and HH on cognitive performance and during a submaximal exercise test. It was hypothesized that cognitive performance was not different between NH and HH. It was further hypothesized that all physiological variables (capillary- and tissue oxygenation, cardiovascular, lactate and cortisol) and subjective variables (perceived exertion, dyspnea, sleep quality, response to hypoxia and task load index) would be different between NH and HH1 and between NH and HH2. It was also hypothesized that there will be a difference between HH1 and HH2 for all physiological and subjective variables, due to acclimatization processes.

## Methods

### Participants

**Fig 1:**
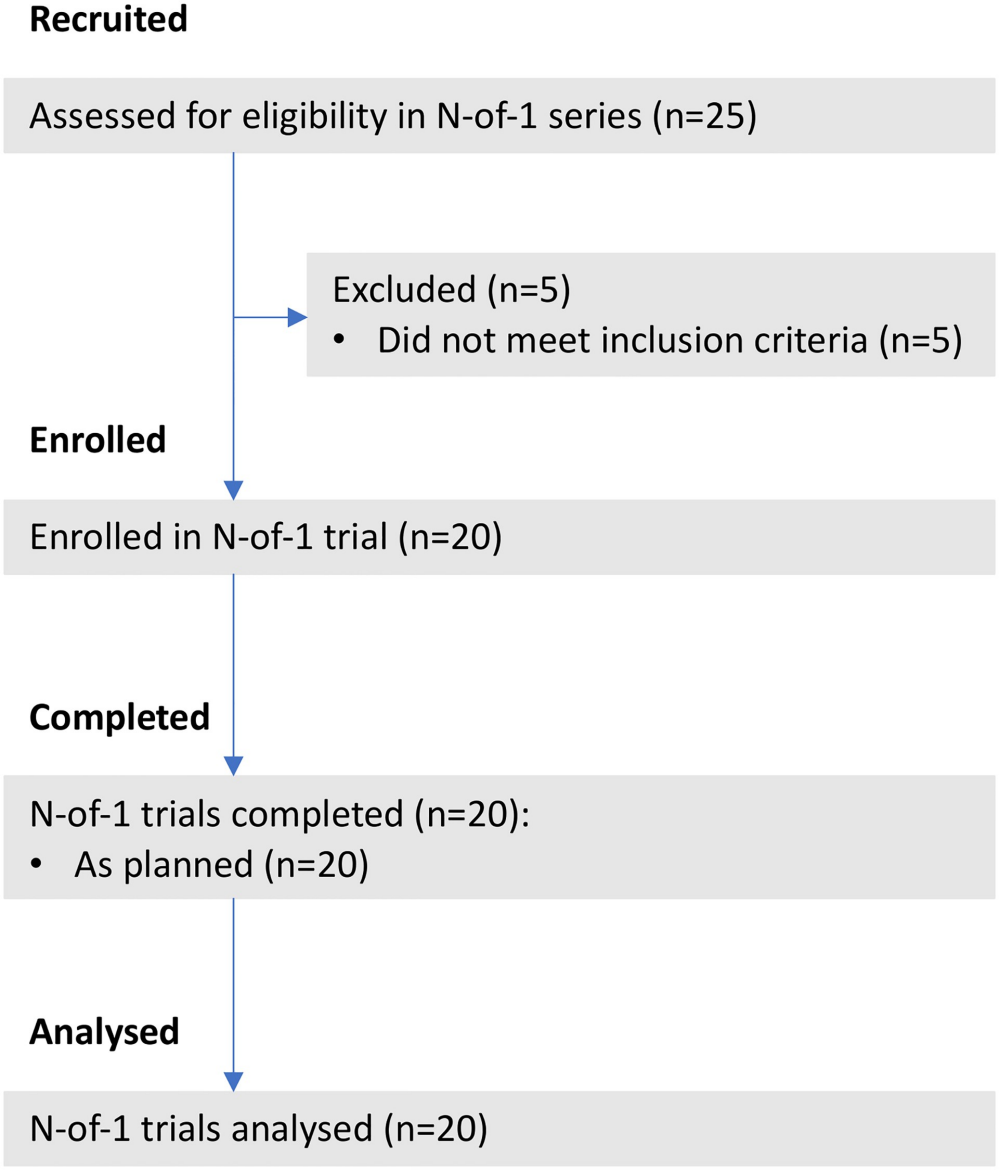
Flow diagram showing the process for participant screening, enrolment and analyses for the study.

A convenience sample of 20 (n = 10 females, 24.8±5.1 yrs, 62.8±6.4 kg, 168.6±6.2 cm; and n = 10 males, 30.3±6.3 yrs, 84.5±9.8 kg, 182.1±5.4 cm) healthy participants volunteered to take part in this study (Fig 1). Females were in the same menstrual phase (identified using a menstrual cycle calendar) or on oral contraception (active pill phase) in the different environments, to minimize hormonal fluctuation. Pilot testing revealed that a total amount of n = 20 participants is feasible for this study to perform the testing in terrestrial altitude in a given time.

All participants were healthy, non-smokers, recreationally trained, and free of any known cardiovascular, respiratory, or neurological disorders. Participants were excluded if they were pregnant, exposed to altitude >1000 m (including commercial flights) for at least one month before the measurements, or if they had ever experienced any altitude-related negative symptoms.

For 72 hours preceding each of the experimental trials, participants were instructed to refrain from strenuous exercise, mentally exhaustive tasks, alcohol, energy drinks, and any other substances that might influence their physiological or cognitive performance. Participants were instructed to maintain their normal eating habits during the entire experiment.

### Ethical consideration

This study was approved by the Swiss Ethical Committee of Zurich (project-ID:2019–00504) in accordance with the Declaration of Helsinki (ICH-GCP), and the trial was pre-registered (ClinicalTrial.gov Identifier: NCT04075565). Written informed consent was obtained from all participants prior to undertaking the experiment.

### Study design

This study employed a partially randomized controlled, crossover design, following the CONSORT 2010 guidelines for randomised crossover trials [38]. Randomization accounts for NN and NH but not for HH1 and HH2. Participants were tested in four experimental environments. In the control environment participants underwent all experimental procedures under laboratory conditions at 550 m (normobaric normoxia, NN, Table 1). Participants were also exposed to simulated altitude (NH) at a F_i_O_2_ of 0.1440. In NH, participants were breathing hypoxic air for 15 min, where baseline measurements were conducted, equivalent to 2980 m using a hypoxic generator (Cloud 9, sporting edge UK LTD, Basingstoke, UK) within the same laboratory. For the hypobaric hypoxia (HH) environment, participants spent two days (HH1, HH2) at terrestrial altitude (2980 m) with one overnight stay (from HH1 to HH2). The time needed to transport the participants from the laboratory (550 m) to the mountain hut (2980 m) was ∼135 min. The baseline was assessed during a 15 min period in all conditions, after which the cognitive performance test and submaximal step-test were conducted. The rationale for including HH2 was to explore the impact of 24 hours of HH compared to the other acute exposures (NH and HH1).

**Table 1:** Environmental conditions (means±SD, n = 20).

	Normobaric normoxia	Normobaric hypoxia	Hypobaric hypoxia day 1	Hypobaric hypoxia day 2	p-value
P_i_O_2_ (mmHg)	146.0±1.5^#+‡^	100.9±1.3*^+‡^	105.6±0.4*^#^	106.0±0.5*^#^	<0.001
F_i_O_2_ (fraction)	0.2093^#^	0.1440*^+‡^	0.2093^#^	0.2093^#^	<0.001
P_B_ (mmHg)	718.1±7.1^+‡^	721.3±9.4^+‡^	522.4±1.0*^#^	523.8±1.1*^#^	<0.001
PH_2_O (mmHg)	20.7±0.8^+‡^	20.3±0.7^+‡^	18.0±2.0*^#^	17.3±2.0*^#^	<0.001
RT (°C)	22.7±0.6^+‡^	22.4±0.6^+‡^	20.5±1.9*^#^	19.8±2.0*^#^	<0.001
RH (%)	39.1±4.3	39.5±3.9	38.2±1.9	38.7±1.3	= 0.621

N = 20, one-way ANOVA, P_i_O_2_, inspired oxygen tension, F_i_O_2_, fraction of inspired oxygen, P_B_, barometric pressure, PH_2_O, water vapor pressure, RT, room temperature, RH, relative humidity. Post-hoc: *p<0.001 to NN, #p<0.001 to NH, +p<0.001 to HH1, ‡ p<0.001 to HH2.

The experiment was conducted over three months, to assure the females to be tested in the same phase of the menstrual cycle (identified by using the calendar counting method). A washout period of a minimum of one week was used between environments. Participants were blinded to the NN and NH environment. In NN, participants wore the mask associated with the hypoxic generator but the tubes were not connected. In HH1 and HH2, participants were also tested whilst wearing the same mask, also without being connected to a hypoxic generator. The mask system was used in all conditions for blinding purposes (NN and NH) and for creating a comparable methodological set-up. The participants didn’t report any breathing difficulties throughout the experiment. All participants underwent an exit questionnaire, which confirmed the successful blinding procedure between NN and NH (p<0.05 identified the correct condition). The environmental conditions are summarized in Table 1.

### Study overview

During their first visit, participants were familiarized with all experimental procedures (cognitive performance test, submaximal step-test). Before each trial, participants were instrumented. Then in an attempt to match the hypoxic dose, participants rested in a seated position for 15 min (breathing normoxic air in NN and hypoxic air in NH and in HH) where baseline data were collected. The reason for the short 15-minute hypoxic exposure for the baseline measurement was that all participants had to be tested within one day at terrestrial altitude (HH1 and HH2) and there would not have been enough time to extend the hypoxic exposure. During the 15-min period, baseline heart rate (HR), HRV, SpO_2_, cerebral oxygenation, and muscle oxygenation were recorded (all data averaged over this period for the analyses). Participants were instructed to minimize talking and movement throughout the baseline measurement period. Mean arterial blood pressure (MAP) and lactate levels were recorded at the end of the 15 min period as a point measurement for the analyses. Then the Groningen sleep quality scale (GSQS), ratings of perceived exertion (RPE), and breathing difficulty (dyspnea) were also completed (all methods described later). Once baseline data collection was completed, the participants performed the cognitive performance battery to assess the cognitive performace, during which the mean cerebral oxygenation was assessed.

Upon completion of the cognitive performance battery, participants performed a 3-min submaximal step-test, as described below. HR, SpO_2_, muscle oxygenation, MAP, lactate, RPE, and dyspnea were collected at the end of the step-test (point measurement) always in the same order in every condition.

Then, participants completed the Lake Louise acute mountain sickness (LLAMS) symptom score to assess the subjective response to hypoxia, followed by an assessment of their task-load index (TLI). In the female cohort, a saliva sample was collected at the end of each day for the assessment of cortisol concentration.

**Fig 2:**
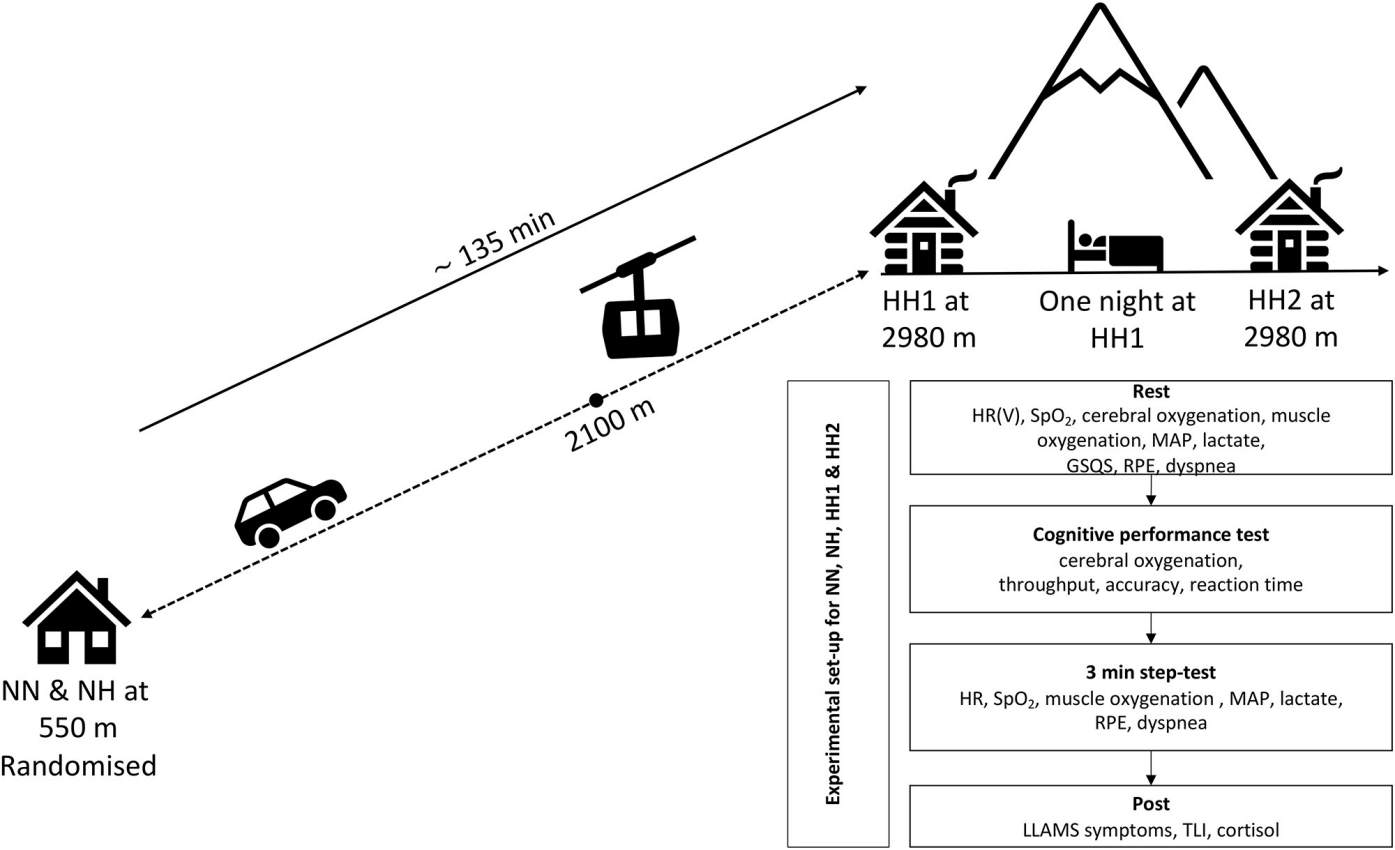
Description of the experimental set-up.

An overview of the experimental setup is provided in Fig 2.

### Tasks

#### Cognitive performance test

Cognitive function was assessed using the Automated Neuropsychological Assessment Metrics (ANAM, VistaLifeSciences Inc., Washington D.C., USA) software on a laptop computer (Lenovo, Hongkong, China) in a seated position.

The validity and the test-retest reliability of this software have been described in detail elsewhere [39]. The battery was designed to examine executive function. Specifically, the battery included tasks associated with concentration, attention, reaction time, memory, processing speed, and decision making. The battery consisted of the following tasks: 2-choice reaction time test, logical relations, manikin, mathematical processing, switching, tower puzzle and the running memory test (n-back 1-, 2-, and 3). These tasks are described in detail elsewhere [39–41].

Before the commencement of testing, participants completed 5 familiarization trials in accordance with the manufacturer’s guidelines. Before each task, an additional practice test was incorporated where the participants had to meet the corresponding cut-off values to start the real task. Participants were instructed to respond to all tests as fast and accurately as possible. For each task, throughput, accuracy, and reaction time of correct responses were recorded. Throughput score is a measure of cognitive efficiency [42] and was considered the main outcome measure for cognitive performance. For the tower puzzle task, the total mean score was used for the analysis.

Participants were instructed to minimize talking and were not verbally influenced by the investigators. Ambient noises were minimized and controlled (Dezibel X, SkyPaw Co. Ltd, Hanoi, Vietnam) in every environment. Cerebral oxygenation was assessed during the test and averaged over time for the analyses.

#### Submaximal step-test

After finishing the cognitive performance test, participants completed a 3-min submaximal step-up test. The test required participants to step-up and down at a rate of 96 steps^.^min^-1^ on a 35 cm step with the assistance of a metronome. A similar submaximal protocol has been used in both normoxic and hypoxic environments [43]. HR, SpO_2_, muscle oxygenation, MAP, lactate, RPE, and dyspnea data were recorded in this order immediately after the completion of the step-task (point measurement).

### Objective measures

#### Oxygenation measurements

SpO_2_ was assessed from the left index finger using a non-invasive (fiber optic) pulse oximeter (Nonin 7500, Nonin medical b.v., Amsterdam, Netherlands).

Cerebral and muscle oxygenation were examined using a calibrated near-infrared spectroscopy device (moorVMS-NIRS, moor instruments, Millwey, United Kingdom) as previously described [19, 44]. The device uses the established spatially resolved spectroscopy technique to measure absolute concentrations of oxygenated, deoxygenated, and total hemoglobin in human tissue [45, 46]. The system measures oxygenation using probes that are placed in contact with the skin. Each probe consists of a detector head that contains two identical photodiodes and an emitter head with two near-infrared LEDs emitting light at approximately 750 nm and 850 nm. Different probe separation holders (30 mm and 50 mm) were used for sampling from different tissue beds, where wider separation offers deeper penetration. Oxygenated (OxyHb), deoxygenated (DeOxyHb) and total hemoglobin (TotHb) were collected in absolute values and expressed in arbitrary units. Tissue oxygenation index (TOI) was calculated (OxyHb / TotHb x 100) and expressed as a percentage value. After each measurement, the reusable probe holder was disinfected before a double-faced adhesive plastic foil was again attached to the probe. Then the probes were inserted into the probe holder and placed on the corresponding skin areas. To avoid interferences from sunlight, the probes were protected with adhesive tape (Hypafix, BSN medical GmbH, Hamburg, Germany).

For cerebral (cTOI, cOxyHb, cDeOxyHb, cTotHb) and muscular (mTOI, mOxyHb, mDeOxyHb, mTotHb) oxygenation, the 50 mm and 30 mm separation probe holder were used, respectively. After the corresponding skin areas were cleaned with an alcohol wipe, the probes were attached to the skin surface of the left prefrontal cortex and the right vastus lateralis (m. quadriceps femoris) as previously described [19, 47]. Near-infrared spectroscopy technology has been demonstrated to be a valid and reliable method to measure tissue oxygenation [48]. SpO_2_ and muscle oxygenation were assessed at baseline (mean over time for 15 min) and at the end of the step-up test (point measurement) whilst cerebral oxygenation was assessed at baseline (mean over time for 15 min) and during (mean over the individual time needed to finish the test) the cognitive performance test.

#### Heart rate and heart rate variability

The evaluation of HR(V) was performed using a polar watch (Polar, V800, Kempele, Finland) and a Bluetooth chest belt (Polar, H10, Kempele, Finland). The Polar V800 monitor has also been demonstrated to be a valid tool for the measurement of R-R intervals during exercise [49]. HRV was collected in a quiet environment over a standardized period of 15 min, where the participants rested in a seated position. The Kubios HRV Premium HRV analysis software, (version 3.4.3.) with the “automatic beat correction” function (Kubios Oy, Kuopio, Finland) was used to analyse the R-R interval data. Parasympathetic nervous system index (PNSi), sympathetic nervous system index (SNSi), and low frequency (LF) to high frequency (HF) ratio (LF/HF ratio) mean and standard deviations were calculated as previously described [50, 51]. HR was assessed at baseline and the end of the submaximal step test (point measurement).

#### Mean arterial pressure

Blood pressure was measured using an automated sphygmomanometer monitor (Beurer BM77, Beurer GmbH, Ulm, Germany) from the left brachial artery. MAP was calculated using the following formula [52]:

MAP = diastolic blood pressure + (systolic blood pressure—diastolic blood pressure) / 3. Blood pressure was assessed at the end of the 15 min period at baseline and the end of the step-up test (both point measurements).

#### Lactate measurement

Blood lactate concentrations were measured using a hand-held lactate analyser (Lactate Scout+, EKF diagnostics, Barleben, Germany). After cleaning the finger with an alcohol wipe, a capillary blood sample (0.2 μl) was taken and analysed. Lactate measurements were conducted at the end of the 15 min baseline period and the end of the step-up test (both point measurements)

#### Cortisol measurement

Salivary cortisol was assessed from the n = 10 female participants to assess the daily stress level after each experimental day [53]. For logistical reasons, only the female participants’ salivary cortisol levels were measured. The saliva was taken from the participants in the evening, between 8:30 pm and midnight, before going to bed. Through a straw, 3mL of saliva was collected and stored in a refrigerator until the analyses were performed the next day using the enzyme-linked immunosorbent assay (ELISA) method [54]. The cortisol-free in saliva ELISA kit is a solid phase enzyme-linked immunosorbent assay, based on the principle of competitive binding. The microtiter wells are coated with a polyclonal rabbit antibody directed against the cortisol molecule. The samples are dispensed in the coated wells and incubated with the enzyme conjugate (cortisol conjugated to horseradish peroxidase). During incubation of endogenous cortisol of a patient, the sample competes with the enzyme conjugate for binding to the coated antibody. The unbound conjugate is removed by washing the wells. Subsequently, the substrate solution is added and the colour development is stopped after a defined time. The intensity of the colour formed is inversely proportional to the concentration of cortisol in the sample. The absorbance is measured at 450 nm with a microtiter plate reader. Salivary cortisol measurements have been demonstrated to be reliable and valid in recreationally active participants [55].

### Subjective measures

#### Sleep quality

Sleep quality of the night before the experimental day was assessed using the validated and reliable GSQS, consisting of a 16-item true or false questionnaire [56] to account for potential influence on cognitive performance [57]. GSQS scores ranged from 0 to 16 whereas higher scores indicated lower subjective sleep quality. Participants were instructed not to leave any item blank and to check the most correct responses. Sleep quality was assessed before the cognitive performance test.

#### Ratings of perceived exertion

RPE was assessed using a 6–20 BORG scale, where participants were asked to call a number (from 6 = no exertion to 20 = maximum exertion) concerning their overall physically perceived exertion. BORG’s RPE has been demonstrated to be a valid tool for monitoring exercise intensity, independent of sex, age and exercise modality, and physical activity level [58]. RPE was assessed at rest and the end of the submaximal step-up test (3-min).

#### Dyspnea

A 0–10 modified BORG scale for dyspnea was used to assess the participants’ breathing status. A number of 0 indicated no breathlessness at all, while 10 indicated maximum breathlessness. The modified BORG scale has been demonstrated to be a valid and reliable tool [59].

Dyspnea was assessed at rest and the end of the submaximal step-up test (3-min).

#### Lake Louise acute mountain sickness symptoms

LLAMS symptoms were assessed at the end of each experimental measurement. The score ranges from 0–15 points and comprised five sections (headache, gastrointestinal symptoms, fatigue and/or weakness, dizziness/light-headedness, and the acute mountain sickness clinical functional score). The rationale for assessing LLAMS symptoms is because of the recommendation to assess acute mountain sickness only after 6 hours, to avoid confusing acute mountain sickness with confounding symptoms (e.g. travelling, vagal response) [60]. Therefore, it was not intended to diagnose AMS but to assess subjective health symptoms, related to hypoxia. LLAMS symptom score was assessed after performing the 3-min step-test (post, Fig 2).

#### Task load index

Subjective workload assessment was performed using the NASA TLX (V.1.0.3., National Aeronautics and Space Administration, Ames Research Center, Moffett, USA) after the submaximal step-test (post, Fig 2). The incorporation of this tool allowed an overall workload score, based on a weighted average of ratings on six subscales (mental demand, physical demand, temporal demand, performance, effort, and frustration).

### Statistical analysis

The assumption of normality was assessed using the Shapiro-Wilk test for small sample sizes. To compare cerebral oxygenation (cTOI, cOxyHb, cDeOxyHb, cTotHb), data were averaged during rest and the cognitive tests. Main and interaction effects were analysed using a two-way analysis of variance (ANOVA) (environment [NN, NH, HH1, HH2] x activity [rest, cognitive performance]).

To compare physiological data (HR, SpO_2_, mTOI, mOxyHb, mDeOxyHb, mTotHb, MAP, lactate) and subjective ratings (RPE, dyspnea) recorded at baseline and at the end of the exercise test, a two-way ANOVA (environment [NN, NH, HH1, HH2] x activity [rest, exercise]) was used. Where necessary, post-hoc analyses were conducted using the least significant difference. Effect sizes are expressed as partial eta squared (η^2^_partial_) values, with 0.1, 0.3, and 0.5 being considered as small, medium, and large, respectively [61]. Analyses of environmental (NN, NH, HH1, HH2) differences for GSQS, HRV (PNSi, SNSi, LF/HF ratio), throughput, accuracy, reaction time, LLAMS symptoms, and TLI were performed using a one-way ANOVA. The statistical analyses were performed using SPSS statistics V. 26 (IBM Corp., Armonk, USA), with the significance level set at p<0.05. Results are presented as mean ± standard deviation unless otherwise stated.

## Results

### Cognitive performance test

#### Cognitive performance outcomes

No significant differences in throughput, accuracy, and reaction time were observed (all p>0.05, Table 2).

**Table 2:** Cognitive performance values (means±SD).

	Normobaric normoxia	Normobaric hypoxia	Hypobaric hypoxia day 1	Hypobaric hypoxia day 2	p-value
**2-choice RT**					
*Throughput*	*156*.*6±17*.*3*	*152*.*8±12*.*2*	*156*.*7±11*.*6*	*159*.*0±10*.*4*	*0*.*64*
*Accuracy (%)*	*98*.*9±1*.*6*	*98*.*5±1*.*6*	*97*.*2±3*.*7*	*98*.*6±1*.*6*	*0*.*13*
*React*. *time (ms)*	*408±58*	*398±36*	*402±49*	*390±48*	*0*.*71*
**Logical relations**					
*Throughput*	*36*.*7±14*.*8*	*34*.*4±13*.*5*	*32*.*3±12*.*5*	*38*.*1±14*.*3*	*0*.*85*
*Accuracy (%)*	*88*.*1±13*.*7*	*88*.*2±14*.*6*	*87*.*0±14*.*9*	*90*.*5±14*.*5*	*0*.*89*
*React*. *time (ms)*	*1775±708*	*1783±619*	*1790±748*	*1638±582*	*0*.*87*
**Manikin**					
*Throughput*	*65*.*9±22*.*1*	*66*.*7±21*.*4*	*57*.*8±22*.*1*	*70*.*3±16*.*3*	*0*.*18*
*Accuracy (%)*	*95*.*0±7*.*4*	*94*.*6±4*.*8*	*95*.*0±4*.*8*	*98*.*0±2*.*0*	*0*.*13*
*React*. *time (ms)*	*1002±376*	*946±322*	*1048±396*	*844±189*	*0*.*24*
**Math. proc.**					
*Throughput*	*25*.*9±8*.*7*	*28*.*2±8*.*7*	*25*.*7±6*.*7*	*29*.*9±7*.*8*	*0*.*31*
*Accuracy (%)*	*91*.*0±8*.*8*	*90*.*5±8*.*7*	*91*.*8±6*.*7*	*92*.*1±8*.*0*	*0*.*91*
*React*. *time (ms)*	*2301±629*	*2122±513*	*2272±594*	*1992±426*	*0*.*25*
**Switching**					
*Throughput*	*39*.*5±11*.*7*	*39*.*7±10*.*1*	*39*.*7±10*.*9*	*43*.*5±10*.*1*	*0*.*11*
*Accuracy (%)*	*97*.*2±2*.*6*	*95*.*5±2*.*9*	*95*.*4±2*.*8*	*96*.*4±2*.*7*	*0*.*15*
*React*. *time (ms)*	*1619±412*	*1543±353*	*1525±340*	*1330±211*	*0*.*05*
**n-back 1**					
*Throughput*	*111*.*4±31*.*5*	*114*.*2±33*.*9*	*100*.*8±32*.*5*	*115*.*8±32*.*3*	*0*.*28*
*Accuracy (%)*	*96*.*8±3*.*9*	*96*.*8±3*.*8*	*96*.*4±4*.*1*	*98*.*0±2*.*2*	*0*.*55*
*React*. *time (ms)*	*693±251*	*564±150*	*616±173*	*539±123*	*0*.*05*
**n-back 2**					
*Throughput*	*60*.*4±24*.*5*	*63*.*5±28*.*9*	*59*.*9±34*.*4*	*71*.*4±28*.*0*	*0*.*30*
*Accuracy (%)*	*89*.*4±11*.*3*	*95*.*2±5*.*1*	*90*.*7±9*.*5*	*94*.*8±6*.*6*	*0*.*10*
*React*. *time (ms)*	*974±403*	*1001±326*	*1050±358*	*897±296*	*0*.*56*
**n-back 3**					
*Throughput*	*53*.*9±31*.*8*	*53*.*5±24*.*3*	*48*.*1±28*.*3*	*49*.*4±23*.*1*	*0*.*80*
*Accuracy (%)*	*81*.*6±12*.*4*	*85*.*2±11*.*4*	*83*.*1±9*.*8*	*84*.*6±9*.*0*	*0*.*71*
*React*. *time (ms)*	*1053±371*	*1100±362*	*1156±366*	*1053±410*	*0*.*86*

N = 20, one-way ANOVA. React. time, reaction time, math. proc., mathematical processing.

For the tower puzzle task, the total mean score was not significantly different between the environments (p>0.05).

#### Cerebral oxygenation

**Fig 3:**
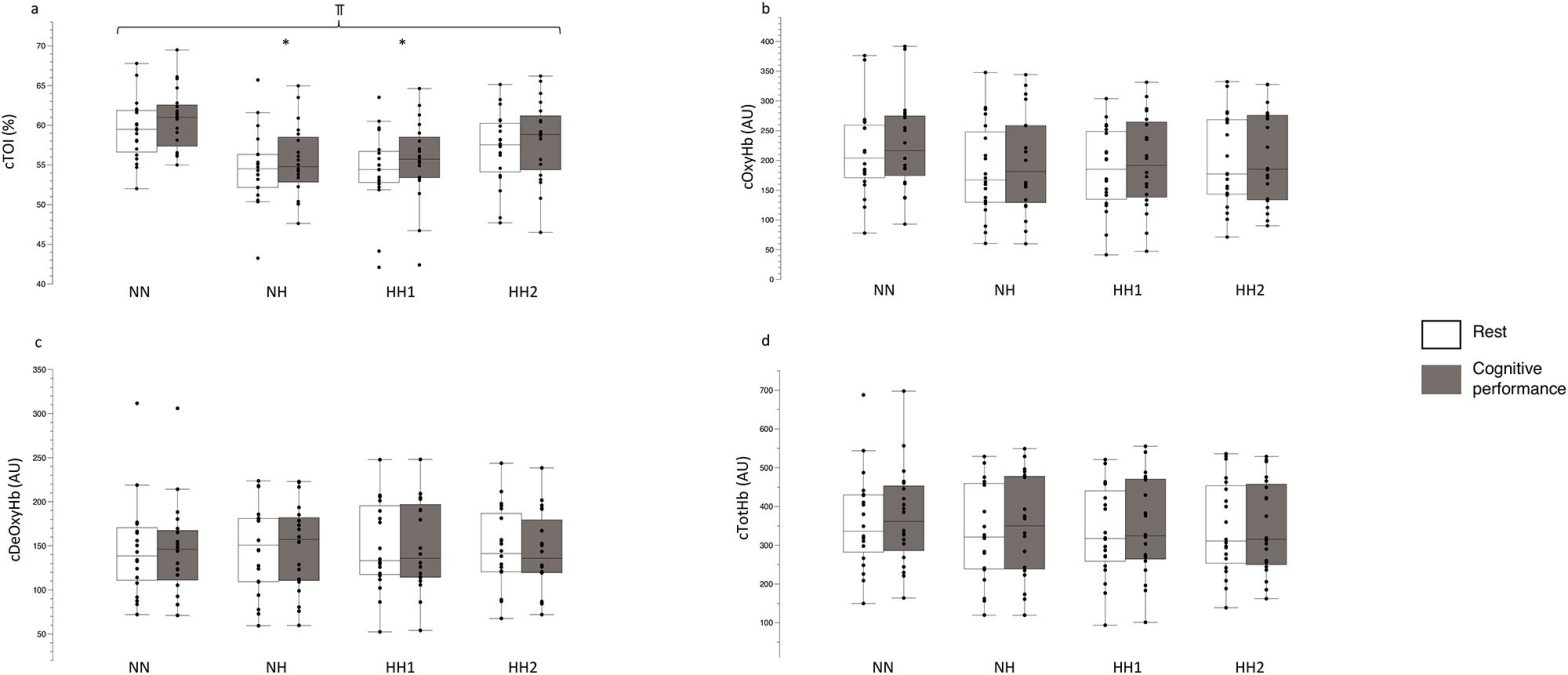
Results for cTOI (a), cOxyHb (b), cDeOxyHb (c), cTotHb (d) at rest and during the cognitive performance test. Values demonstrate the median, iqr, min, max values. Dots indicate the individual values of each participant. ⫪ p = 0.001 main difference between environments, * p = 0.001 to NN.

A significant main effect for both environment (F_3,76_ = 5.84, p = 0.001, η^2^_partial_ = 0.18, Fig 3a) and activity (F_1,76_ = 40.45, p<0.001, η^2^_partial_ = 0.34), but not a environment*activity interaction (F_3,76_ = 0.47, p = 0.69, η^2^_partial_ = 0.01) was observed in cTOI. There were no differences between the hypoxic (NH vs HH1, NH vs HH2, HH1 vs HH2) conditions for cTOI values (all p>0.05).

The cTOI values were only higher (p = 0.001) in NN (rest: 59.4±3.8%, cognitive performance: 60.7±3.8%) compared to NH (rest: 54.6±4.6%, cognitive performance: 55.4±4.5%; ΔcTOI NN vs NH: 5.03±1.4%) and higher (p = 0.001) in NN compared to HH1 (HH1 rest: 54.3±4.9%, HH1 cognitive performance: 55.6±5.1%; ΔcTOI NN vs HH1: 5.14±1.4%).

There were no main effects for environment (all: p>0.05) for cOxyHb (F_3,76_ = 0.74, η^2^_partial_ = 0.02, Fig 3b), cDeOxyHb (F_3,76_ = 0.05, η^2^_partial_ = 0.002, Fig 3c) and cTotHb (F_3,76_ = 0.23, η^2^_partial_ = 0.009, Fig 3d).

### Submaximal step-test

#### Capillary and muscular oxygenation

**Fig 4:**
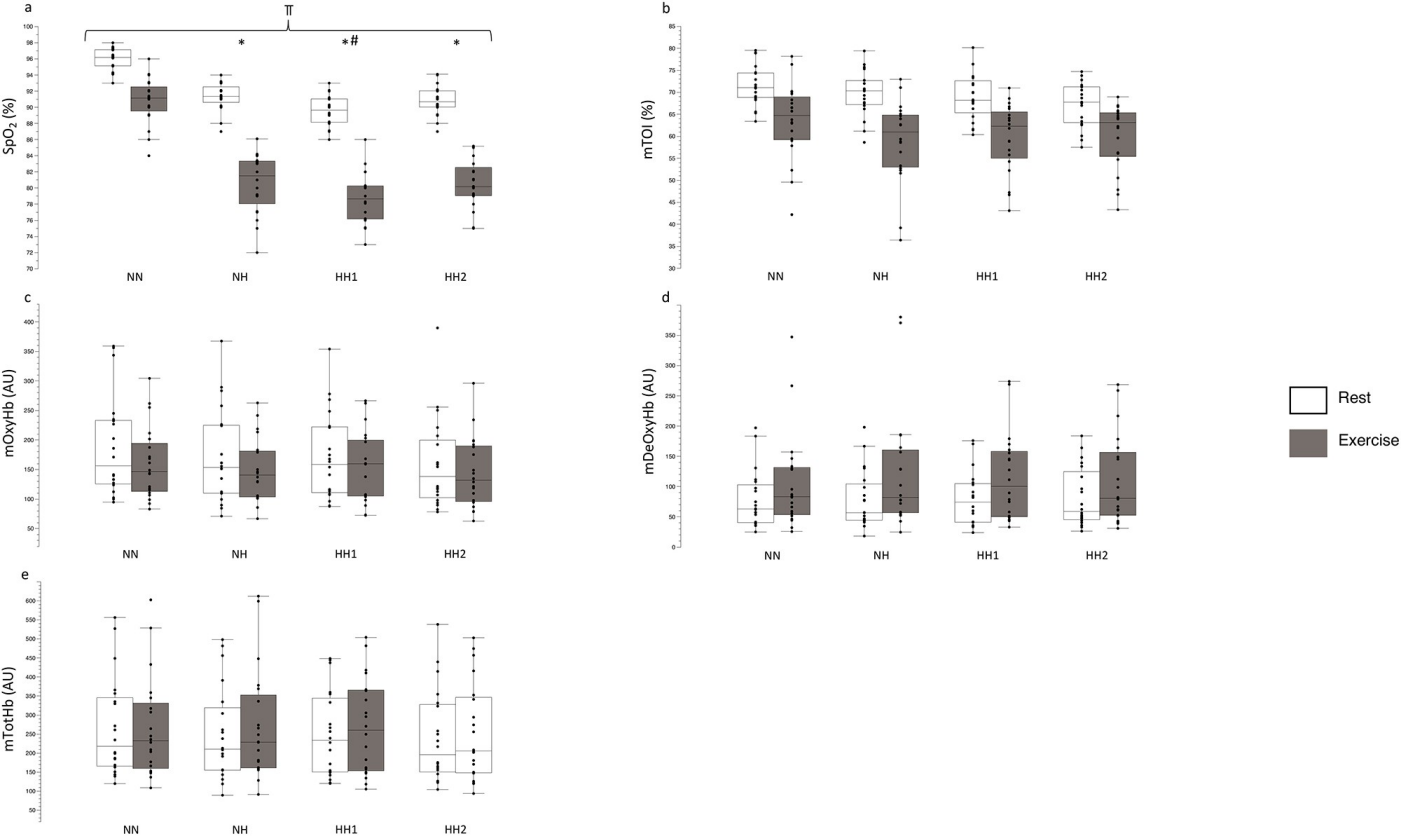
Results for SpO_2_ (a), mTOI (b), mOxyHb (c), mDeOxyHb (d), mTotHb (e) at rest and at the end of the 3-min step-test (exercise). Values demonstrate the median, iqr, min, max values. Dots indicate the individual values of each participant. ⫪ p<0.001 main difference between environments, * p<0.001 to NN, # p = 0.008 to NH.

A significant main effect for environment (F_3,76_ = 101.0, p<0.001, η^2^_partial_ = 0.80, Fig 4a), and activity (F_1,76_ = 534.4, p<0.001, η^2^_partial_ = 0.87), and their interaction (F_3,76_ = 12.2, p<0.001, η^2^_partial_ = 0.32) was observed in SpO_2_.

In NH, SpO_2_ was higher compared to HH1 (ΔSpO_2_ NH vs HH1: 1.7±0.5%, p = 0.003). No differences were detected between NH compared to HH2 and HH1 vs HH2 (both p>0.05).

SpO_2_ was higher (p<0.001) in NN (rest: 95.8±1.2%, 3-min step-test: 90.7±2.8) vs NH (rest: 91.3±1.7%, 3-min step-test: 80.5±3.6%, ΔSpO_2_ NN vs NH: 7.3±0.5%). The SpO_2_ values were also higher (p<0.001) in NN vs HH1 (HH1 rest: 89.5±1.8%, HH1 3-min step-test: 78.7±3.1%, ΔSpO_2_ NN vs HH1: 9.1±0.5%) and higher (p<0.001) in NN vs HH2 (HH2 rest: 90.7±1.8%, HH2 3-min step-test: 80.5±3.0%, ΔSpO_2_ NN vs HH2: 7.6±0.5%).

For NN, ΔSpO_2_ was only 5.0±0.7% (p<0.001) between rest and at the end of the 3-min step-test. In NH, ΔSpO_2_ was 10.7±0.7% (p<0.001), at HH1, it was 10.8±0.7% (p<0.001) and at HH2, ΔSpO_2_ was 10.2±0.7% (p<0.001) between rest and the end of the 3-min step-task.

For muscle oxygenation, no main differences between the environments (all: p>0.05) were observed for mTOI (F_3,76_ = 0.21, η^2^_partial_ = 0.05, Fig 4b), mOxyHb (F_3,76_ = 0.76, η^2^_partial_ = 0.01, Fig 4c), mDeOxyHb (F_3,76_ = 0.98, η^2^_partial_ = 0.002, Fig 4d) and mTotHb (F_3,76_ = 0.97, η^2^_partial_ = 0.003, Fig 4e).

#### Cardiovascular response

The cardiovascular values can be observed in Table 3.

**Table 3:** Cardiovascular response to submaximal exercise.

	Normobaric normoxia	Normobaric hypoxia	Hypobaric hypoxia day 1	Hypobaric hypoxia day 2	p-value
**HR (bpm)**	# ‡	* ‡	‡	* # +	
*Rest*	71.0±10.9	77.6±10.2	75.0±9.6	86.0±11.2	0.001
*3-min step-test*	137.4±12.9	141.6±10.4	141.9±10.6	144.9±5.8	
**MAP (mmHg)**					
*Rest*	*94*.*0±8*.*1*	*93*.*6±8*.*3*	*97*.*4±8*.*8*	*98*.*1±7*.*7*	*0*.*28*
*3-min step-test*	*116*.*8±12*.*7*	*114*.*7±11*.*8*	*110*.*6±28*.*0*	*122*.*1±10*.*5*	
**Lactate (mmol/l)**					
*Rest*	*1*.*7±0*.*6*	*2*.*0±0*.*9*	*2*.*4±0*.*8*	*2*.*3±0*.*8*	*0*.*57*
*3-min step-test*	*4*.*7±2*.*4*	*5*.*0±1*.*4*	*4*.*9±1*.*5*	*4*.*8±1*.*6*	

N = 20, repeated-measures ANOVA.

* p<0.05 to NN,

^#^ p<0.05 to NH,

^+^ p<0.05 to HH 1,

^‡^ p<0.05 to HH2.

The analyses demonstrated that HR was significantly different between environments (F_3,76_ = 5.91, p = 0.001, η^2^_partial_ = 0.18, Table 3) with a significant activity (F_1,76_ = 2274.09, p<0.001, η^2^_partial_ = 0.98) and environment*activity interaction (F_3,76_ = 1.85, p<0.001, η^2^_partial_ = 0.06).

Specifically, HR values were lower (p = 0.03) in NH vs HH2 (ΔHR NH vs HH2: 5.8±2.6 bpm) and also lower (p = 0.01) in HH1 (rest: 75.0±9.6 bpm, 3-min step-test: 141.9±10.6 bpm) vs HH2 (ΔHR HH1 vs HH2: 6.9±2.6 bpm).

The HR was also lower (p = 0.04) in NN (rest: 71.0±10.9 bpm, 3-min step-test: 137.4±12.9 bpm) vs NH (rest: 77.6±10.2 bpm, 3-min step-test: 141.6±10.4 bpm, ΔHR NN vs NH: 5.4±2.6 bpm). HR was lower (p<0.001) in NN vs HH2 (HH2 rest: 86.0±11.2 bpm, HH2 3-min step-test: 144.9±5.8 bpm, ΔHR NN vs HH2: 11.2±2.6 bpm).

For MAP and lactate, no significant main effect of environment (both p>0.05) was observed.

#### Ratings of perceived exertion & dyspnea

No main effect of environment (p>0.05, η^2^_partial_ = 0.01) was observed for RPE (ΔNN vs NH: -0.03±0.3, ΔNN vs HH1: -0.33±0.3, ΔNN vs HH2: -0.16±0.3) and dyspnea (p>0.05, η^2^_partial_ = 0.01) (ΔNN vs NH: -0.10±0.2, ΔNN vs HH1: -0.21±0.2, ΔNN vs HH2: -0.18±0.2).

### Autonomic nervous system and stress response

#### Heart rate variability

**Fig 5:**
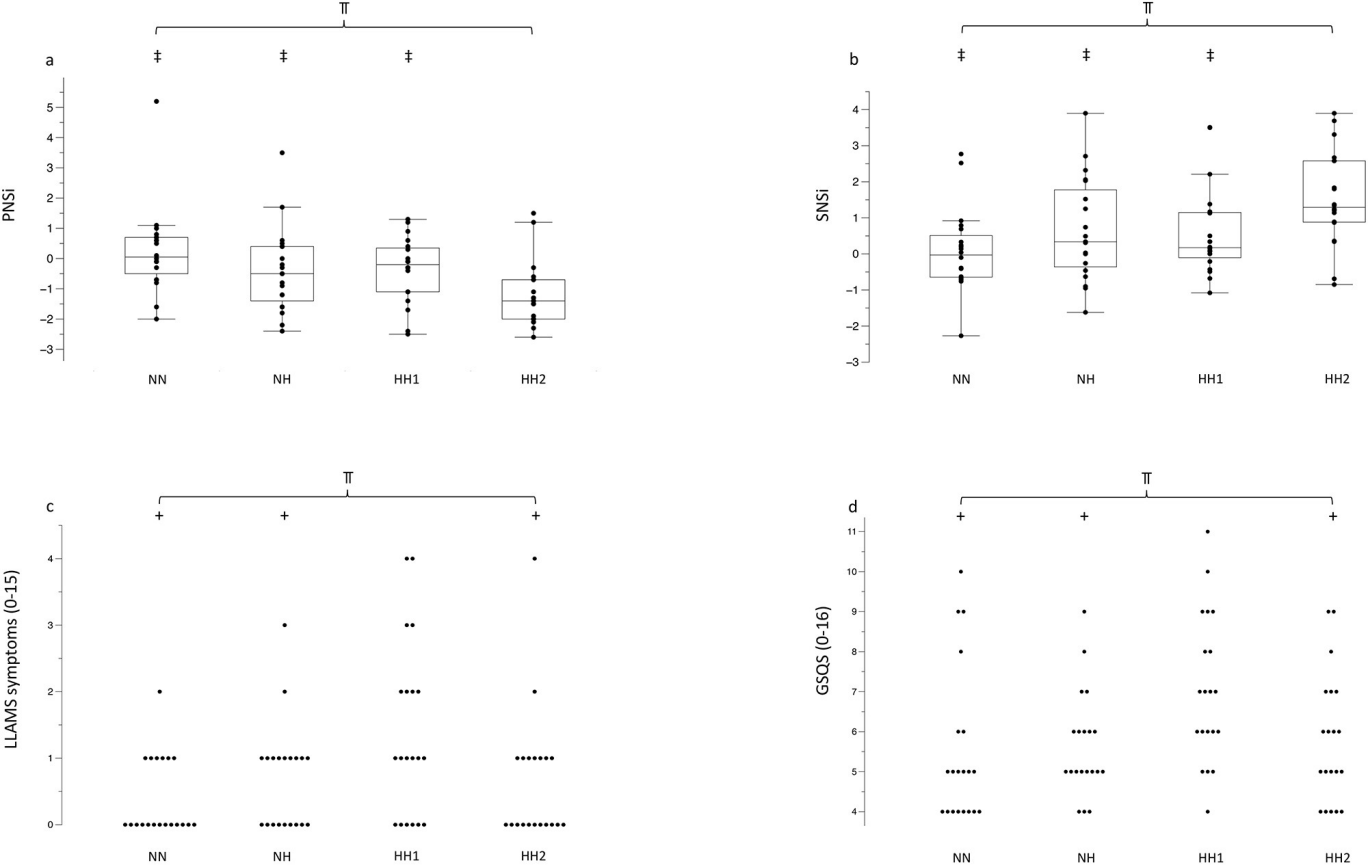
Results for PNSi (a), SNSi (b), LLAMS symptoms (c) using the LLAMS score, sleep quality ratings using the GSQS (d). Values demonstrate the median, iqr, min, max values in 4a and 4b. Dots indicate the individual values of each participant. ⫪ p<0.05 main difference between environments, + p<0.05 to HH1, ‡ p<0.05 to HH2.

PNSi was different (p = 0.006, η^2^_partial_ = 0.15) between environments at rest (Fig 5a).

PNSi was higher (p = 0.04) in NH compared to HH2 (-0.40±1.39 vs. -1.20±1.05) and higher (p = 0.032) in HH1 compared to HH2 (-0.33±1.05 vs. -1.20±1.05).

PNSi was also higher (p<0.001) in NN compared to HH2 (0.24±1.43 vs. -1.20±1.05).

SNSi was significantly different (p = 0.006, η^2^_partial_ = 0.14) between environments (Fig 5b). Specifically, SNSi was also lower (p = 0.03) in NH vs. HH2 (0.66±1.40 vs. 1.54±1.29) and lower (p = 0.02) in HH1 compared to HH2 (0.58±1.25 vs. 1.54±1.29).

SNSi was also lower (p = 0.001) in NN compared to HH2 (0.07±1.12 vs. 1.54±1.29).

The LF/HF ratio was not different (p = 0.13) between environments.

#### Salivary cortisol

Salivary cortisol was 0.23±0.19 μg/l in NN, 0.24±0.16 μg/l in NH and at HH1 and HH2 0.31±0.19 μg/l and 0.25±0.12 μg/l, with no differences between environments (p = 0.72, η^2^_partial_ = 0.39).

### Self-perceived ratings

#### Lake Louise acute mountain sickness symptoms

For LLAMS symptom score, a significant difference (p = 0.010, η^2^_partial_ = 0.13) between environments was detected (Fig 5c). Participants reported more (p = 0.001) symptoms in HH1 (1.4±1.3) compared to NN (0.4±0.5), more (p = 0.014) symptoms in HH1 compared to HH2 (0.6±0.9) and also more (p = 0.01) symptoms in HH1 compared to NH (0.6±0.7, Fig 5c).

#### Sleep quality

A significant difference in sleep quality (p = 0.02, η^2^_partial_ = 0.12, Fig 5d) was observed between environments. Lower sleep quality was reported on the night before HH1 (7.0±1.8) compared to NN (5.5±1.9, p = 0.005), NH (5.6±1.3, p = 0.011), and HH2 (5.8±1.6, p = 0.023).

#### Task load index

There was no difference between environments for any TLI’s subscale (mental demand, physical demand, temporal demand, performance, effort and frustration, p>0.05 for all).

## Discussion

Using a partially randomized controlled, crossover design, this study investigated the effects of both NH and HH (HH1) on cognitive performance, physiological and perceptual responses and also compared them to an overnight stay in HH (HH2).

The findings of this study demonstrate no differences between the hypoxic conditions for cognitive performance outcomes and cerebral oxygenation during the cognitive performance battery. There were also no differences detected between the hypoxic conditions for muscle oxygenation, mean arterial pressure, lactate concentrations, and subjective responses during the exercise test.

However, our findings also show significant differences between normobaric and hypobaric hypoxia. In NH, SpO_2_ remains higher compared to HH1, whilst heart rate (during exercise) and sympathetic activation (at rest) are both lower in NH compared to HH2. Differences are also detected between the two days in hypobaric hypoxia. In HH1, heart rate and sympathetic activation are lower compared to HH2. Sleep quality was lower, and LLAMS symptoms are higher in HH1 compared to HH2.

The effects of hypoxia on cognitive performance outcomes are often contradictorily within the literature. Authors have demonstrated no impairment in cognitive performance at extremely high altitudes during ascend to the Mt. Everest [62], whilst others showed very specific cognitive impairments at milder (±4300 m) levels of altitude [63]. Another potential rationale for these contradictory findings is the methodological heterogeneity in the assessment of cognitive function across the literature [19]. Using a meta-analytical approach, McMorris and colleagues have suggested that low (35–60 mmHg) PaO_2_ is the key predictor of cognitive performance (R_2_ = 0.45, p < 0.001) and this was independent of whether the exposure was in NH or HH [17]. Williams and colleagues (2019) have also shown that reductions in peripheral oxygen saturation and cerebral oxygenation are responsible for a decrease in cognitive performance during NH [19]. Specifically, these authors demonstrated that accuracy in a n-back task, a measure of working memory, was only impaired after a 60-min exposure to a F_i_O_2_ of 0.12 and 0.145, but not in 0.17, which compared to NN. Ochi and colleagues (2018) have also shown a decline in the performance of the Stroop task (i.e. a complex central executive task) in a F_i_O_2_ of 0.105, but not in 0.135 or 0.165 [64]. Moreover, there was a negative correlation reported between Stroop interference and SpO_2_ [64]. Although the exact aetiology or physiological mechanism(s) responsible for a reduction in cognitive performance following hypoxia have yet to be fully elucidated, several confounding variables, including duration and severity of the hypoxic exposure, ambient temperature, blinding, and inter-individual variability have to be taken into account [65].

By design and as expected cTOI was lower in NH (ΔcTOI NN vs NH: 5.03±1.4%) and HH1 (ΔcTOI NN vs HH1: 5.14±1.4%) compared to NN (Fig 3a). These data are similar to those reported where cTOI was investigated following similar NH and HH exposures [66]. In this study, the potential differences in cerebral oxygenation under altitude (3450 m) vs hypoxia under normobaric (485 m) were evaluated. Although the terrestrial altitude was around 400m higher compared to our study, no differences between NH and HH were observed [66]. However, it has to be considered that in this study, positive expiratory pressure breathing was conducted, leading to improved cerebral oxygenation values in NH and HH. Another study investigated the difference between NH and HH on cerebral oxygenation even higher, at a (simulated) altitude of 3883 m, demonstrating reduced values within conditions but found also no between-condition differences [3]. Interestingly, cTOI values were not different in HH2 compared to NN in our study, which may suggest that some minor phenotypic adaptations may have occurred in HH, as discussed elsewhere [67]. In addition, similar to previously published research [11], SpO_2_ was higher in NH compared to HH1 (ΔSpO_2_ NH vs HH1: 1.7±0.5%, Fig 4a) in our study.

Although large inter-individual variability was observed in our study, PNSi (Fig 5a) was lower, whilst SNSi (Fig 5b) and heart rate (Table 3) tended to be higher in HH2 compared to NH and HH1. Similar variability, both in physiological and perceptual responses, has previously been observed [23]. The present findings are similar to those of others investigating the differences between NH and HH [23, 24]. Oxygen saturation was around 4% lower in HH compared to NH [24] whereas the difference was smaller in our study at a (simulated) lower altitude. SpO_2_ was also significantly lower in another study in HH compared to NH, which was primarily attributed to the increase in dead space ventilation due to the P_B_ reduction [23]. In addition, these authors found also reduced end-tidal partial pressure of O_2_ and CO_2_ in HH compared to NH [23, 24] which might explain the differences. This mechanism might have occurred because the ambient partial pressure of nitrogen is initially lower than the body’s and therefore nitrogen initially diffuses from the tissues to the alveoli [6]. Until this equilibrium is achieved, arterial O_2_ and CO_2_ content are lowered as a result of the relatively higher partial pressure of alveolar nitrogen in HH vs NH [9]. This stresses the importance of carrying out studies within a certain time frame (hypoxic dose), as potential differences between NH and HH might be related to hypoxic exposure times.

Hypoxaemia has been demonstrated to be a potent activator of the sympathoadrenal system [68, 69]. In the present study, the second day at altitude (HH2) led to the most pronounced changes in the autonomic nervous system (Figs 5a and 5b). It has been concluded that the sympathetic tone remains increased and parasympathetic tone remains decreased during acute hypoxia and that during acclimatization, a progressive shift towards a still higher sympathetic tone occurs [70]. A possible explanation might be that peripheral chemoreceptors can act as regulators of the autonomic activity and reset baroreflex control of heart rate and sympathetic activity, allowing higher levels of heart rate, blood pressure, and sympathetic drive [71]. In line with our study, others have also demonstrated a sympathetic predominance in HH, at a comparable altitude of 2980 m, compared to NH [27]. However, HRV was assessed using R-R intervals in the current study and this method is known to be influenced by breathing frequency following hypoxic exposure [70, 72].

As reported in the methods section, our intention was not to assess AMS because several hours of exposure to hypoxia are needed to induce this symptom complex [73]. However, during the first day of HH, symptoms of fatigue and/or weakness and gastrointestinal symptoms were higher compared to the other hypoxic conditions. This finding corroborates others who have investigated the differences between HH and NH with a comparable F_i_O_2_ of 0.142 [74]. However, the most rated symptoms in our HH1 condition were fatigue and/or weakness and gastrointestinal symptoms. It is therefore thinkable, that higher symptoms were detected at HH1 because of the reduced sleeping hours in this condition, which occurred due to transportation reasons. The gastrointestinal problems (e.g. poor appetite or nausea) might also be a result of getting up earlier on the first day of travelling to the mountain.

In our study, sleep quality was lowest in HH1 compared to NH but also compared to HH2. However, it has to be taken into account, that sleep quality was assessed always from the night before the experiments were carried out. Consequently, the reduced sleep quality in HH1 might be attributed to the fact, that the participants had to wake up earlier to be transported to the mountain. The impaired sleep quality can’t be directly linked to hypoxia in our study.

This study was not without limitations. First, the hypoxic exposure was modest and it’s plausible that a more severe hypoxic stimulus may have led to different findings. Regardless, this HH environment was selected in particular because this altitude is commonly visited by most of the population during leisure time and tourists in the alps, resulting in a large societal relevance. Second, although we attempted to match P_i_O_2_ between the environments, there was a statistical, albeit small difference of ∼6mmHg between the NH and the HH environments. Although we attempted to match the hypoxic dose between normobaric and hypobaric hypoxia as precisely as possible, exact timing was not possible due to external (travelling time to the mountain hut) and technical (limited control of room environment on the mountain, working hours of the cable car) reasons. Third, although a relatively small convenience sample of n = 20 participants was recruited for this study, this sample size compares favourably to the mean number of participants (i.e., n = 12) in a systematic review comparing normobaric and hypobaric hypoxia [9]. This is perhaps reflective of the logistical challenges associated with conducting a study involving a terrestrial altitude and a controlled laboratory crossover design.

## Conclusion

In conclusion, only SpO_2_ was higher in NH compared to HH1. Heart rate and sympathetic activation remained higher in HH2 compared to NH and HH1, whilst subjective symptoms to hypoxia were highest in HH1 compared to all other conditions.

These results account for a P_i_O_2_ between 100–106 mmHg. Comparable results between normobaric- and hypobaric hypoxia were found for cognitive performance and cerebral oxygenation. There were also no differences between hypoxic conditions for muscular oxygenation, mean arterial pressure and lactate concentrations. The observed physiological variations in individual responses to hypoxia indicate that future studies should focus on unravelling the underlying mechanisms of normobaric- and hypobaric hypoxia on human physiology. Another perspective for future studies would be to extend the comparison at lower P_i_O_2_ levels.

## Supporting information

S1 Checklist (DOCX)

## Abbreviations

cDeOxyHb: cerebral deoxygenated haemoglobin
cOxyHb: cerebral oxygenated haemoglobin
cTOI: cerebral tissue oxygenation index
cTotHb: cerebral total haemoglobin
F_i_O_2_: fraction of inspired oxygen
GSQS: Groningen sleep quality scale
HH: hypobaric hypoxia
HR: heart rate
HRV: heart rate variability
LLAMS: Lake Louise acute mountain sickness
MAP: mean arterial blood pressure
mDeOxyHb: muscular deoxygenated haemoglobin
mOxyHb: muscular oxygenated haemoglobin
mTOI: muscular tissue oxygenation index
mTotHb: muscular total haemoglobin
NH: normobaric hypoxia
NN: normobaric normoxia
PaO_2_: partial pressure of arterial oxygen
P_B_: barometric pressure
PH_2_O: water vapour pressure
P_i_O_2_: partial pressure of inspired oxygen
PNSi: parasympathetic nervous system index
RH: relative humidity
RPE: ratings of perceived exertion
RT: room temperature
SNSi: sympathetic nervous system index
SpO_2_: arterial oxygen saturation
TLI: task load index
