# Supplementary material for: The effects of normobaric and hypobaric hypoxia on cognitive performance and physiological responses: A crossover study
Source: PLoS One. 2022 Nov 10;17(11):e0277364. doi: 10.1371/journal.pone.0277364 (PMC9648783; doi:10.1371/journal.pone.0277364)
Supplement: S1 Checklist — (DOCX) [file pone.0277364.s001.docx]

**Table 1** CENT 2015 checklist*****; CONSORT 2010 checklist items with modifications or additions for individual or series of N-of-1 trials; empty items in the CENT 2015 column indicate no modification from the CONSORT 2010 item

| Section/  Topic | CONSORT 2010 | |  | CENT 2015 | |
| --- | --- | --- | --- | --- | --- |
|  | No | Item |  | No | Item |
| Title and abstract | | |  | | |
|  | 1a | Identification as a randomised trial in the title |  | 1a | Identify as an “N-of-1 trial” in the title  ***For series:*** Identify as “a series of N-of-1 trials” in the title |
|  | 1b | Structured summary of trial design, methods, results, and conclusions (for specific guidance see CONSORT for abstracts) |  | 1b | *For specific guidance, see CENT guidance for abstracts (table 2)* |
| Introduction | | |  | | |
| Background and objectives | 2a | Scientific background and explanation of rationale |  | 2a.1 |  |
|  |  |  |  | 2a.2 | Rationale for using N-of-1 approach |
|  | 2b | Specific objectives or hypotheses |  | 2b |  |
| Methods | | |  | | |
| Trial design | 3a | Description of trial design (such as parallel, factorial) including allocation ratio |  | 3a | Describe trial design, planned number of periods, and duration of each period (including run-in and wash out, if applicable)  ***In addition for series:*** Whether and how the design was individualized to each participant, and explain the series design |
|  | 3b | Important changes to methods after trial start (such as eligibility criteria), with reasons |  | 3b |  |
| Participant(s) | 4a | Eligibility criteria for participants |  | 4a† | Diagnosis or disorder, diagnostic criteria, comorbid conditions, and concurrent therapies.  ***For series:*** Same as CONSORT item 4a |
|  | 4b | Settings and locations where the data were collected |  | 4b† |  |
|  |  |  |  | 4c | Whether the trial(s) represents a research study and if so, whether institutional ethics approval was obtained |
| Interventions | 5 | The interventions for each group with sufficient details to allow replication, including how and when they were actually administered |  | 5 | The interventions for each period with sufficient details to allow replication, including how and when they were actually administered |
| Outcomes | 6a | Completely defined pre-specified primary and secondary outcome measures, including how and when they were assessed |  | 6a.1 |  |
|  |  |  |  | 6a.2 | Description and measurement properties (validity and reliability) of outcome assessment tools |
|  | 6b | Any changes to trial outcomes after the trial commenced, with reasons |  | 6b |  |
| Sample size | 7a | How sample size was determined |  | 7a |  |
|  | 7b | When applicable, explanation of any interim analyses and stopping guidelines |  | 7b |  |
| Randomisation: |  |  |  |  |  |
| Sequence generation | 8a | Method used to generate the random allocation sequence |  | 8a | Whether the order of treatment periods was randomised, with rationale, and method used to generate allocation sequence |
|  | 8b | Type of randomisation; details of any restriction (such as blocking and block size) |  | 8b | When applicable, type of randomisation; details of any restrictions (such as pairs, blocking) |
|  |  |  |  | 8c | Full, intended sequence of periods |
| Allocation concealment mechanism | 9 | Mechanism used to implement the random allocation sequence (such as sequentially numbered containers), describing any steps taken to conceal the sequence until interventions were assigned |  | 9 |  |
| Implementation | 10 | Who generated the random allocation sequence, who enrolled participants, and who assigned participants to interventions |  | 10 |  |
| Blinding | 11a | If done, who was blinded after assignment to interventions (for example, participants, care providers, those assessing outcomes) and how |  | 11a |  |
|  | 11b | If relevant, description of the similarity of interventions |  | 11b |  |
| Statistical methods | 12a | Statistical methods used to compare groups for primary and secondary outcomes |  | 12a | Methods used to summarize data and compare interventions for primary and secondary outcomes |
|  | 12b | Methods for additional analyses, such as subgroup analyses and adjusted analyses |  | 12b | ***For series:*** If done, methods of quantitative synthesis of individual trial data, including subgroup analyses, adjusted analyses, and how heterogeneity between participants was assessed, (for specific guidance on reporting syntheses of multiple trials, please consult the PRISMA Statement) |
|  |  |  |  | 12c | Statistical methods used to account for carryover effect, period effects, and intra-subject correlation |
| Results | | |  | | |
| Participant flow (a diagram is strongly recommended) | 13a | For each group, the numbers of participants who were randomly assigned, received intended treatment, and were analysed for the primary outcome |  | 13a.1 | Number and sequence of periods completed, and any changes from original plan with reasons |
|  |  |  |  | 13a.2 | ***For series:*** The number of participants who were enrolled, assigned to interventions, and analysed for the primary outcome |
|  | 13b | For each group, losses and exclusions after randomisation, together with reasons |  | 13c | ***For series:*** losses or exclusions of participants after treatment assignment, with reasons, and period in which this occurred, if applicable |
| Recruitment | 14a | Dates defining the periods of recruitment and follow-up |  | 14a† |  |
|  | 14b | Why the trial ended or was stopped |  | 14b | Whether any periods were stopped early and/or whether trial was stopped early, with reason(s). |
| Baseline data | 15 | A table showing baseline demographic and clinical characteristics for each group |  | 15† |  |
| Numbers analysed | 16 | For each group, number of participants (denominator) included in each analysis and whether the analysis was by original assigned groups |  | 16 | For each intervention, number of periods analysed.  ***In addition for series:*** if quantitative synthesis was performed, number of trials for which data were synthesized |
| Outcomes and estimation | 17a | For each primary and secondary outcome, results for each group, and the estimated effect size and its precision (such as 95% confidence interval) |  | 17a.1 | For each primary and secondary outcome, results for each period; an accompanying figure displaying the trial data is recommended. |
|  |  |  |  | 17a.2 | For each primary and secondary outcome, the estimated effect size and its precision (such as 95% confidence interval)  ***In addition for series:*** if quantitative synthesis was performed, group estimates of effect and precision for each primary and secondary outcome |
|  | 17b | For binary outcomes, presentation of both absolute and relative effect sizes is recommended |  | 17b |  |
| Ancillary analyses | 18 | Results of any other analyses performed, including subgroup analyses and adjusted analyses, distinguishing pre-specified from exploratory |  | 18 | Results of any other analyses performed, including assessment of carryover effects, period effects, intra-subject correlation  ***In addition for series:*** If done, results of subgroup or sensitivity analyses |
| Harms | 19 | All important harms or unintended effects in each group (for specific guidance see CONSORT for harms) |  | 19 | All harms or unintended effects for each intervention. *(for specific guidance see CONSORT for harms)* |
| Discussion | | |  | | |
| Limitations | 20 | Trial limitations, addressing sources of potential bias, imprecision, and, if relevant, multiplicity of analyses |  | 20 |  |
| Generalisability | 21 | Generalisability (external validity, applicability) of the trial findings |  | 21 |  |
| Interpretation | 22 | Interpretation consistent with results, balancing benefits and harms, and considering other relevant evidence |  | 22 |  |
| Other information | | |  | | |
| Registration | 23 | Registration number and name of trial registry |  | 23 |  |
| Protocol | 24 | Where the full trial protocol can be accessed, if available |  | 24 |  |
| Funding | 25 | Sources of funding and other support (such as supply of drugs), role of funders |  | 25 |  |

*****It is strongly recommended that this checklist be read in conjunction with the CENT 2015 Explanation and Elaboration^24^ for important clarification on the items. The copyright for CENT (including checklist) is held by the CENT Group and is distributed under a Creative Commons Attribution (CC-BY 4.0) license.

†Caution should be taken when reporting potentially identifying information pertaining to CENT items 4a, 4b, 14a, and 15.
